# Syncope Associated with Subthalamic Nucleus Deep Brain Stimulation in a Patient with Parkinson's Disease

**DOI:** 10.1155/2013/371929

**Published:** 2013-12-22

**Authors:** Dursun Aygun, Ersoy Kocabicak, Onur Yildiz, Musa Kazim Onar, Hatice Guz, Omer Boke, Murat Kurt, Yasin Temel

**Affiliations:** ^1^Department of Neurology, Ondokuz Mayis University, Samsun, Turkey; ^2^Department of Neurosurgery, Faculty of Medicine, Ondokuz Mayis University, 55200 Samsun, Turkey; ^3^Department of Neurosurgery and Neuroscience, Maastricht University Medical Center, P. Debyelaan 25, 6202 AZ Maastricht, The Netherlands; ^4^Department of Psychology, Ondokuz Mayis University, Samsun, Turkey; ^5^Department of Psychiatry, Ondokuz Mayis University, Samsun, Turkey

## Abstract

In advanced Parkinson's disease (PD), deep brain stimulation (DBS) may be an alternative option for the treatment of motor symptoms. Side effects associated with subthalamic nucleus (STN) DBS in patients with PD are emerging as the most frequent sensory and motor symptoms. DBS-related syncope is reported as extremely rare. We wanted to discuss the mechanisms of syncope associated with STN DBS in a patient with Parkinson's disease. *Case report.* Sixty-three-year-old female patient is followed up with diagnosis of idiopathic Parkinson's disease for 6 years in our clinic. The patient has undergone STN DBS due to painful dystonia and drug resistant tremor. During the operation, when the left STN was stimulated at 5 milliampere (mAmp), the patient developed presyncopal symptoms. However, when the stimulation was stopped symptoms improved. During the early period after the operation, when the right STN was stimulated at 1.3 millivolts (mV), she developed the pre-yncopal symptoms and then syncope. Our case shows that STN DBS may lead to directly autonomic symptoms resulting in syncope during stimulation-on (stim-on).

## 1. Introduction

Parkinson's disease (PD) is a chronic neurodegenerative disorder primarily characterized by progressive motor impairments due to dopamine insufficiency. As the disease progresses, levodopa-related motor complications develop. At this stage, it is difficult to treat motor symptoms of PD. In advanced PD, deep brain stimulation (DBS) may be an alternative option for the treatment of motor symptoms. Side effects associated with subthalamic nucleus (STN) DBS in patients with PD are not rare. These side effects are emerging as the most frequent sensory and motor symptoms [1]. Also STN DBS is associated with neuropsychiatric side effects and weight gain in some individuals. DBS related syncope is reported as extremely rare. The pathomechanisms of these side effects are completely unknown. However, it has been suggested that these side effects are associated with the spread of electrical activity to the structures surrounding the STN [1]. We wanted to discuss the mechanisms of syncope associated with STN DBS in a patient with Parkinson's disease.

## 2. Case Report

Sixty-three-year-old female patient is followed with diagnosis of idiopathic PD since 6 years in our clinic. The patient has undergone STN DBS due to painful dystonia and drug resistant tremor. The patient's clinical motor symptoms were assessed by UPDRS III in both off and on medication states. Thus, levodopa response (LR) rate of the patient was determined. During the operation, the STN regions with the highest amplitude via electrophysiological recordings from the right and left STN were determined. Then, to determine the permanent contacts on each electrode, each target was tested in terms of the best motor benefits clinically and minimal side effects. The patient was examined by a movement disorders specialist neurologist (DA) during these tests which are made in increments of 1 mAmp voltage. After all, the right electrode was placed within center of the STN and the left electrode was placed within the anterior part of the STN. During the operation, when the anterior part (target −1) of the left STN was stimulated at higher voltages (5 mAmp), the patient developed presyncopal symptoms. However, when the stimulation was stopped symptoms improved. During the early period after the operation, when voltage applied to the electrode (target +2) placed within the right STN was increased from 1.1 mV to 1.3 mV, the patient developed the presyncopal symptoms and then syncope. The stimulation was immediately stopped and the patient improved. Syncope lasted less than 1 min, and the patient's right STN voltage was reduced to 1.1 mV. The latest, the right electrode, which is at ventral of the STN, has been stimulated as monopolar by 2,1 mV, and the left electrode has been stimulated as bipolar by 3 mV. Preoperative clinical and demographic characteristics of the patient are shown in Table 1.

## 3. Discussion

In PD, DBS related syncope is extremely rare. One study including all neuropsychiatric patients with DBS reported that syncope was 9,3% of DBS presentations to the emergency department (ED) [2]. However, it is not easy to prove the relationship between DBS and syncope in the ED. Kenney et al. reported that of 319 DBS cases, including 182 PD patients, eight (2.5%) developed vasovagal response and four (1.2%) developed syncope [3]. Our case may the first report as a detailed presentation of DBS-related syncope case in the literature. To our knowledge, there is a few DBS-related syncope or orthostatic hypotension case reported for PD in the literature. Williams et al. reported a PD case with worsened orthostatic hypotension due to STN DBS [4]. In a study of 14 PD patients with STN-DBS, three patients showed orthostatic hypotension during stim-on, two in stim-off, and three during both conditions [5]. This study suggested that STN DBS has some selective positive effects on autonomic functions without any effect on the cardiovascular autonomic nervous system (ANS). It is known that there are connections with the limbic system of the STN. It has been reported that as the basal ganglia are connected to areas involved in regulation of the ANS, STN DBS may affect ANS functions [5]. Also the STN is adjacent to the lateral side of the hypothalamus. The STN has functionally four sections. Benedetti et al. suggested that the dorsal STN is involved in autonomic control [6]. That is, its mediodorsal part has been linked with the limbic system. For this reason, it may lead to the autonomic symptoms to stimulate the medial-rostral part of the STN, adjacent to the hypothalamus. In contrast, Greenhouse et al. suggested that there has been a possible role of the ventral sector of the STN in limbic function [7].

## 4. Conclusion

Our case shows that STN DBS may lead to directly autonomic symptoms resulting in syncope during stim-on. This status may be due to stimulation of the connecting part with the limbic system of the STN or/and to spread of stimulation to the hypothalamus.

## Figures and Tables

**Table 1:** Preoperative clinical and demographic characteristics of the patient.

Characteristics	
Age (Year)	63
Sex	Female
Profession	Farmer
Education	Illiterate
Living area	Rural
MMSE (ON)	29
Subtype	Tremor dominant
Disease duration (Year)	5.5
Levodopa response (%)	63.1
Hoehn and Yahr (OFF)	3
FLASQ-PD (C + D + E)	28
ADL (OFF; %)	60
Off time (% of waking day)	25
Dyskinesia (% of waking day)	26–50
Total dopaminergic medication	1350 mg

MMSE: The minimental state examination; FLASQ-PD: The Florida Surgical Questionnaire for PD; ADL: Schwab and England activities of daily living.
